# Risk factors and prediction model for chronic bacterial infection in stable bronchiectasis in Shanghai, China

**DOI:** 10.3389/fmed.2026.1900247

**Published:** 2026-09-01

**Authors:** Yuxian Chen, Shaoyan Zhang, Ben Su, Rui Zhou, Tao Chen, Xinyuan Xu, Zhengyi Zhang, Dingzhong Wu, Zhenhui Lu, Lei Qiu

**Affiliations:** Institute of Respiratory Diseases, Longhua Hospital, Shanghai University of Traditional Chinese Medicine, Shanghai, China

**Keywords:** bronchiectasis, chronic bacterial infection, nomogram, prediction model, risk factors, stable phase

## Abstract

**Background:**

Chronic bacterial infection (CBI) represents a key feature in patients with bronchiectasis. Therefore, it is of great clinical significance to develop an effective nomogram model for predicting the risk of CBI in stable bronchiectasis, which guides individualized clinical treatment strategies.

**Methods:**

The study enrolled patients in stable bronchiectasis in Shanghai between January 2020 and December 2024. They were categorized into two groups of CBI and without CBI. We used Univariate logistic analysis, LASSO regression and Multivariate logistic analysis to identify predictors associated with CBI. Based on the screened-out risk factors, a nomogram was constructed to predict the risk of CBI in adults with stable bronchiectasis. We used receiver operating characteristic, the area under the curve (AUC) and calibration curve to determine the predictive accuracy and discriminability of nomogram. The decision curve analysis (DCA) was employed to further confirm the clinical effectiveness of nomogram.

**Results:**

Multivariate logistic analysis revealed the risk factors of CBI included history of smoking, number of lobes affected ≥3, number of exacerbation in the prior year ≥3, history of hemoptysis in the prior year, CRP, CD3^+^CD4^+^T-cell count <500 cells/μL. The AUC was 0.861 (95% CI: 0.825–0.897). We developed a nomogram model. Based on AUC, Hosmer-Lemeshow goodness-of-fit test (*p* = 0.794), calibration curve, and DCA, we conducted that the model exhibits excellent predictive accuracy, discriminability and clinical effectiveness.

**Conclusion:**

The nomogram model demonstrated satisfactory discrimination and calibration accuracy, enabling screen patients with stable bronchiectasis at high risk of CBI and facilitating individualized clinical decisions in future clinical practice.

## 1 Introduction

Bronchiectasis is defined as a chronic inflammatory airway disease characterized by abnormal, permanent dilation of the bronchi. Its clinical manifestations include chronic cough, sputum production, and recurrent respiratory infections (1). The prevalence of bronchiectasis has been increasing annually, there is a significantly higher prevalence of bronchiectasis in Asian (76–1,249 per 100, 000) compared to Europe (53–362 per 100, 000 individuals). From 2013 to 2017, the prevalence of bronchiectasis among Chinese adults increased by 2.31-fold, showing a significant upward trend. The prevalence is projected to rise to 448.93 per 100, 000 population by 2030 (2, 3). And the mortality rate of patients with bronchiectasis is significantly higher than that of those without bronchiectasis (4, 5), imposing a substantial burden on both patients and society. The pathogenesis of bronchiectasis remains incompletely understood. The “vicious cycle” hypothesis proposed by *P. J. Flume* et al. summarized the pathophysiological mechanism of bronchiectasis as a “vicious vortex,” which involves the reciprocal interaction and mutual reinforcement of four key components: airway infection, airway inflammation, impaired mucociliary clearance, and structural lung damage (6). Of these factors, infection is the major driver in the development and progression of bronchiectasis (1).

The stable state of bronchiectasis was defined as a clinically stable condition without new symptoms and with no requirement for antibiotics or oral corticosteroids for pulmonary exacerbation in the preceding 4 weeks (7). Definitions of chronic bacterial infection (CBI) were highly heterogeneous, with previous study defining it as two or more positive sputum cultures of the same micro-organism at least 3 months apart over 1 year while in a stable state of bronchiectasis (8). Among patients with stable bronchiectasis, the prevalence of CBI ranges from 45 to 72%, common potential pathogenic microorganisms (PPMs) isolated from respiratory specimens of bronchiectasis patients include *Pseudomonas aeruginosa* (PA), *Haemophilus influenzae*, *Staphylococcus aureus*, and *Streptococcus pneumoniae* (9). CBI leads to persistent airway inflammation, directly impairing lung structure, and affecting airway mucus clearance function. CBI is typically associated with more frequent exacerbations, greater disease severity, and worse clinical outcomes, thereby emphasizing the importance of conducting regular microbiological testing in patients with bronchiectasis (10, 11). Sputum culture is a primary non-invasive method for the routine evaluation of bronchiectasis patients, and all patients are recommended to receive sputum culture examination to monitor respiratory microorganisms load and type (12, 13). Therefore, the identification of high-risk populations with stable bronchiectasis who may suffer from chronic bacterial infection and regular sputum culture testing are of significant clinical value for implementing early intervention, preventing acute exacerbations, and enhancing prognosis and quality of life.

Previous studies mainly focused on the management of acute exacerbations of bronchiectasis. Currently, there is no research on the risk factors and prediction models of CBI in patients with stable bronchiectasis in China. Therefore, we conducted a prospective cohort study at Longhua Hospital Affiliated to Shanghai University of Traditional Chinese Medicine from January 2020 to December 2024. By analyzing the clinical characteristics and screening risk factors, the study aims to develop a nomogram model to identify high-risk populations with CBI among patients with stable bronchiectasis, providing insights for individualized management and early intervention.

## 2 Materials and methods

### 2.1 Study design and participants

A total of 380 patients in the stable phase of bronchiectasis were prospectively enrolled from Longhua Hospital Affiliated to Shanghai University of Traditional Chinese Medicine between January 2020 and December 2024.

The patients we collected must meet the following criteria: (1) individuals between the ages of 18 and 75; (2) bronchiectasis was diagnosed by high-resolution computed tomography (HRCT); (3) patients were in a clinically stable state at enrollment.

The exclusion Criteria were as follows: (1) severe organ dysfunction; (2) co-existing autoimmune diseases or severe immunodeficiency; (3) concurrent malignancy; (4) hospitalization in the preceding 4 weeks; (5) pregnancy or lactation; (6) mental or cognitive impairment; (7) bronchiectasis due to cystic fibrosis; (8) the clinic data is incomplete. Informed consent was obtained from all patients before patient inclusion, and the study was approved by the Medical Ethics Committee of Longhua Hospital (Approval No. 2019LCSY058 and 2024LCSY144).

Bronchiectasis was diagnosed by high-resolution computed tomography (HRCT) scans demonstrating a bronchoarterial ratio >1, lack of tapering, and airways visibility within 1 cm of the pleural surface or touching mediastinal pleura, along with clinical symptoms consistent with bronchiectasis (12). The stable state of bronchiectasis was defined as a clinically stable condition without new symptoms and with no requirement for antibiotics or oral corticosteroids for pulmonary exacerbation in the preceding 4 weeks (7). An acute exacerbation is defined as a deterioration for at least 48 h in three or more of the following key symptoms, coupled with a clinical decision to change the patient’s bronchiectasis treatment: (1) cough, (2) sputum volume and/or consistency, (3) purulence of sputum, (4) dyspnea and/or exercise intolerance, (5) fatigue and/or malaise, (6) hemoptysis (14).

### 2.2 Research methods

Clinical data were systematically collected and organized, encompassing the following domains: (1) Demographics characteristics: sex, age, and body mass index (BMI); (2) Information of disease: history of smoking, history of alcohol using, disease duration, number of exacerbations in the prior year, history of hemoptysis in the prior year, bronchiectasis severity index (BSI) score, E-FACED score; (3) Past medical history: tuberculosis, pneumonia, pertussis, measles, chronic rhinosinusitis, allergic rhinitis, gastroesophageal reflux disease (GERD), diabetes, hypertension, coronary artery disease, liver diseases, kidney diseases, cerebrovascular diseases; (4) Laboratory tests: blood routine examinations, C-reactive protein (CRP), immune function index; (5) Chest HRCT: number of lobes affected, type of bronchiectasis, and the modified Reiff score; (6) Pulmonary function tests: forced vital capacity (FVC), FVC% pred, forced expiratory volume in 1 s (FEV_1_), FEV_1_% pred, the FEV_1_/FVC ratio, and Fractional exhaled Nitric Oxide (FeNO).

Patients were categorized into CBI and without CBI groups based on the presence of chronic bacterial infection during the stable state. Definitions of chronic bacterial infection were highly heterogeneous, with previous study defining it as two or more positive sputum cultures of the same micro-organism at least 3 months apart over 1 year while in a stable state of bronchiectasis (8).

In this study, the BSI score and E-FACED score were adopted to comprehensively evaluate the disease severity of patients with bronchiectasis. The modified Medical Research Council (mMRC) dyspnea scale was used to characterize the severity of dyspnea, and the modified Reiff score was applied to assess the radiological severity of patients with bronchiectasis. The BSI score is primarily utilized to predict future exacerbations, hospital admissions and mortality in patients with bronchiectasis. It is calculated from the following parameters: age (maximum 6 points), BMI (2 points), FEV_1_%pred (3 points), significant hospital admission (5 points), exacerbations in the prior year (2 points), mMRC score (3 points), *Pseudomonas aeruginosa* colonization (3 points), colonization with other organisms (1 point) and number of affected lung lobes on imaging (1 point). The maximum score is 26 points, stratified as mild (score 0–4), moderate (score 5–8) and severe (score ≥ 9) (15). The E-FACED score mainly predicts the risk of future exacerbations and hospital admissions in patients with bronchiectasis. It is calculated from FEV_1_%pred (2 points), age (2 points), *Pseudomonas aeruginosa* colonization (1 point), number of affected lung lobes on imaging (1 point), mMRC score (1 point), and previous hospital admission due to exacerbation (2 point). The maximum score is 9 points, with stratification into mild (score 0–3), moderate (score 4–6) and severe (score 7–9) (16, 17). The modified mMRC score can assess dyspnea severity at different activity levels and consists of five grades (scores ranging from 0 to 5) (18). The modified Reiff score evaluates bronchiectasis according to morphological type (cylindrical: 1 point; varicose: 2 points; cystic: 3 points) and extent (a total of six lung lobes). The maximum score is 18 points, classified as mild (1–6 points), moderate (7–12 points), and severe (13–18 points) (19, 20).

Lymphocyte subset testing was performed via flow cytometry. For whole blood specimens used in detection, the leukocyte concentration should be ≤20 × 10^9^/L with a carryover contamination rate ≤0.5%, and a minimum of 5,000 lymphocytes were acquired per tube. Immunofluorescent staining of whole blood was performed using CD45-containing antibody cocktails to analyze lymphocyte subsets. Lymphocyte populations with purity ≥95% were gated based on CD45/SSC dot plots. Subsequently, the percentages and absolute counts of CD3^+^ T cells, CD4^+^ T cells, CD8^+^ T cells, B cells and NK cells were quantified for each population (21, 22).

### 2.3 Sputum culture

Sputum bacterial cultures were performed in the department of clinical laboratory, Longhua Hospital Affiliated to Shanghai University of Traditional Chinese Medicine. Regarding the sputum samples, patients should rinse oral cavity with clean water or sterile normal saline prior to collection, and expectorate no less than 3 mL of deep-lung sputum into a sterile container through forceful deep coughing. When sputum was not spontaneously expectorated, induction was performed using nebulized hypertonic (5%) saline. Bacterial culture was performed and interpreted by senior laboratory staff. Prior to bacterial culture, quality evaluation was performed on every sputum sample by means of smear microscopy. Samples containing less than 10 squamous epithelial cells per low-power field were regarded as qualified (23, 24). Each qualified specimen was inoculated onto sheep blood agar, chocolate agar, and MacConkey agar by using 4-quadrant streaking method, and incubated at 35 °C for 48 h. The culture results were observed at 24 h and 48 h of incubation. The predominant organisms were identified if only one type of bacterium was present or one bacterial type was present in a greater quantity than others on semiquantitative culture (25). The identification and quantification of predominant organisms were determined based on colony growth observed: growth only in sector 1 was scored as +; growth in sectors 1 and 2 as ++; growth in sectors 1, 2 and 3 with fewer than 5 colonies in sector 3 as +++; growth in sectors 1, 2 and 3 with more than 5 colonies in sector 3 as ++++.

### 2.4 Lung function test

All lung function tests were performed in the pulmonary function laboratory, Longhua Hospital Affiliated to Shanghai University of Traditional Chinese Medicine. Before each measurement, volume calibration was implemented with a 3-L calibration syringe. Flow linearity was verified under low, medium and high flow rates to guarantee the accuracy of the equipment. Spirometry tests were performed using the JAEGER MasterScreen-body + diffusion + APS system (Germany) to evaluate pulmonary function and bronchodilator reversibility (26). All participants remained seated and wore a nasal clip throughout the examination, which was executed 15 min after salbutamol inhalation. Acceptable expiratory maneuvers should be free of interruptions, coughing, and air leaks (27). Moreover, exhalation needed to persist for a minimum of 6 s or until an apparent volume plateau emerged. Every subject completed no less than three valid forced expiratory maneuvers to achieve reproducible outcomes. Recorded parameters encompassed forced vital capacity (FVC), FVC% predicted, forced expiratory volume in one second (FEV₁), FEV₁% predicted, and the FEV₁/FVC ratio. Changes in FVC, FEV₁, FVC% predicted, FEV₁% predicted and FEV₁/FVC ratio were calculated as (the first follow-up measurement) − (baseline measurement).

### 2.5 Statistical analysis

All data analyses were conducted using SPSS software (version 27.0) and R software (version 4.4.2) for Windows. Continuous variables were tested for normality using the Shapiro–Wilk test. Normally distributed continuous variables were expressed as mean ± standard deviation (SD), with inter-group comparisons conducted using independent-samples t-tests. Non-normally distributed continuous variables were presented as median (inter-quartile range, IQR), and inter-group comparisons were assessed by the Mann–Whitney *U* rank-sum test. Categorical variables were presented as frequency and percentage (*n*, %), with inter-group differences evaluated by the chi-square test or Fisher’s exact test.

All variables were subjected to univariate logistic analysis to identify potential risk factors. The variables with a *p*-value﹤0.20 were entered in LASSO regression to mitigate overfitting and select optimal variables through coefficient penalization. Variables identified from LASSO regression were incorporated into the multivariate logistic analysis using the forced entry method to determine independent predictors of CBI. Associations were expressed as odds ratios (OR) and 95% confidence intervals (CIs). Eventually, based on variables with a *p*-value﹤0.05 in multivariate logistic analysis, we developed a nomogram model to predict the risk of CBI.

The nomogram’s discrimination was evaluated by the receiver operating characteristic curve (ROC). AUC value is the area covered by ROC curve. The prediction effect increases as the AUC value get closer to 1. AUC﹥0.70 indicates that the model discrimination is acceptable. The goodness-of-fit of nomogram was assessed using calibration curves, which showed the level of consistency between the predicted values and the actual values. Additionally, the clinical utility of nomogram was estimated by decision curve analysis (DCA), which can compare the difference between nomogram and other model by quantifying the net benefit under different threshold probabilities. In this study, a *p*-value of less than 0.05 (bilateral) was considered statistically significance.

## 3 Results

### 3.1 Baseline clinical characteristics of patients

The study included 380 patients with stable bronchiectasis. The median age of the patients was 64 years. The median BMI was 18.52 kg/m^2^. Most patients were females (63.7%), and 17.4% were current or ex-smokers. Among them, 208 patients (54.74%, 208/380) were classified as the CBI group, while 172 patients comprised the group without CBI. Demographic and clinical characteristics of all patients were comprehensively summarized in Table 1. Among patients with CBI, the detection rate of *Pseudomonas aeruginosa* (PA) was 65.87% (137/208), *Haemophilus influenzae* was 13.46% (28/208), *Streptococcus pneumoniae* was 6.25% (13/208), *Moraxella catarrhalis* was 5.29% (11/208), *Staphylococcus aureus* was 3.37% (7/208), *Klebsiella pneumoniae* was 2.88% (6/208), and *Acinetobacter baumannii* was 2.88% (6/208). The patients in CBI group were older and had a lower BMI compared to the without CBI group. The proportions of patients with smoking and drinking history were significantly higher in the CBI group. In addition, the CBI group exhibited longer disease duration, greater number of lobes affected, higher annual frequency of exacerbations, and more annual hemoptysis. These patients also had higher BSI scores. In terms of laboratory indicators, the CBI group showed elevated CRP levels and decreased counts of CD3^+^CD4^+^ T cells.

**Table 1:** Demographics and clinic characteristics of the 380 patients with stable bronchiectasis stratified by chronic bacterial infection.

Characteristic	Overall *N* = 380	Without CBI *N* = 172	CBI *N* = 208	Statistic	*p*-value
Demographics					
Sex, *n* (%)				*χ*^2^ = 1.96	0.161^1^
Female	242 (63.7%)	103 (59.9%)	139 (66.8%)		
Male	138 (36.3%)	69 (40.1%)	69 (33.2%)		
Age, median (Q1, Q3)	64.00 (53.00, 68.00)	62.50 (49.75, 67.00)	64.00 (55.75, 70.00)	*Z* = −2.74	0.006^2, *^
BMI, median (Q1, Q3)	18.52 (17.34, 19.49)	19.05 (17.85, 20.53)	18.29 (17.12, 19.07)	*Z* = −5.67	<0.001^2, *^
Information of disease					
History of smoking, *n* (%)				*χ*^2^ = 4.59	0.032^1, *^
No	314 (82.6%)	150 (87.2%)	164 (78.8%)		
Yes	66 (17.4%)	22 (12.8%)	44 (21.2%)		
History of alcohol using, *n* (%)				*χ*^2^ = 5.42	0.020^1, *^
No	196 (51.6%)	100 (58.1%)	96 (46.2%)		
Yes	184 (48.4%)	72 (41.9%)	112 (53.8%)		
Disease duration, median (Q1, Q3)	13.00 (8.00, 17.00)	11.00 (5.75, 15.00)	14.00 (9.00, 19.00)	*Z* = −4.92	<0.001^1, *^
Type of bronchiectasis, *n* (%)				*χ*^2^ = 10.33	0.006^1, *^
Cylindrical	227 (59.7%)	118 (68.6%)	109 (52.4%)		
Cystic	49 (12.9%)	18 (10.5%)	31 (14.9%)		
Both of above	104 (27.4%)	36 (20.9%)	68 (32.7%)		
Number of lobes affected, median (Q1, Q3)	3.00 (2.00, 4.00)	2.00 (2.00, 3.00)	3.00 (3.00, 4.00)	*Z* = −7.44	<0.001^2, *^
Exacerbations in the prior year, *n* (%)				*χ*^2^ = 11.88	<0.001^1, *^
No	158 (41.6%)	88 (51.2%)	70 (33.7%)		
Yes	222 (58.4%)	84 (48.8%)	138 (66.3%)		
Number of exacerbations in the prior year, median (Q1, Q3)	1.00 (0.00, 3.00)	0.00 (0.00, 2.00)	2.00 (0.00, 3.00)	*Z* = −6.17	<0.001^1, *^
History of hemoptysis in the prior year, *n* (%)				*χ*^2^ = 24.99	<0.001^1, *^
No	293 (77.1%)	153 (89.0%)	140 (67.3%)		
Yes	87 (22.9%)	19 (11.0%)	68 (32.7%)		
Reiff score, *n* (%)				*χ*^2^ = 1.90	0.386^1^
1–6	300 (78.9%)	141 (82.0%)	159 (76.4%)		
7–12	68 (17.9%)	27 (15.7%)	41 (19.7%)		
13–18	12 (3.2%)	4 (2.3%)	8 (3.8%)		
BSI score, *n* (%)				*χ*^2^ = 32.74	<0.001^1, *^
0–4	164 (43.2%)	98 (57.0%)	66 (31.7%)		
5–8	186 (48.9%)	71 (41.3%)	115 (55.3%)		
≥9	30 (7.9%)	3 (1.7%)	27 (13.0%)		
E-FACED score, *n* (%)				*χ*^2^ = 0.79	0.373^1^
0–3	353 (92.9%)	162 (94.2%)	191 (91.8%)		
4–6	27 (7.1%)	10 (5.8%)	17 (8.2%)		
Past medical history					
Tuberculosis, *n* (%)				*χ*^2^ = 0.08	0.774^1^
No	289 (76.1%)	132 (76.7%)	157 (75.5%)		
Yes	91 (23.9%)	40 (23.3%)	51 (24.5%)		
Pneumonia, *n* (%)				*χ*^2^ = 0.02	0.893^1^
No	317 (83.4%)	143 (83.1%)	174 (83.7%)		
Yes	63 (16.6%)	29 (16.9%)	34 (16.3%)		
Pertussis, *n* (%)				*χ*^2^ = 0.24	0.627^1^
No	360 (94.7%)	164 (95.3%)	196 (94.2%)		
Yes	20 (5.3%)	8 (4.7%)	12 (5.8%)		
Measles, *n* (%)				*χ*^2^ = 0.00	>0.999^3^
No	370 (97.4%)	168 (97.7%)	202 (97.1%)		
Yes	10 (2.6%)	4 (2.3%)	6 (2.9%)		
Chronic sinusitis, *n* (%)				*χ*^2^ = 0.87	0.351^1^
No	329 (86.6%)	152 (88.4%)	177 (85.1%)		
Yes	51 (13.4%)	20 (11.6%)	31 (14.9%)		
Allergic rhinitis, *n* (%)				*χ*^2^ = 0.12	0.729^1^
No	363 (95.5%)	165 (95.9%)	198 (95.2%)		
Yes	17 (4.5%)	7 (4.1%)	10 (4.8%)		
Gastroesophageal reflux disease, *n* (%)				*χ*^2^ = 0.12	0.729^1^
No	363 (95.5%)	165 (95.9%)	198 (95.2%)		
Yes	17 (4.5%)	7 (4.1%)	10 (4.8%)		
Diabetes, *n* (%)				*χ*^2^ = 0.54	0.461^1^
No	278 (73.2%)	129 (75.0%)	149 (71.6%)		
Yes	102 (26.8%)	43 (25.0%)	59 (28.4%)		
Hypertension, *n* (%)				*χ*^2^ = 0.00	0.966^1^
No	247 (65.0%)	112 (65.1%)	135 (64.9%)		
Yes	133 (35.0%)	60 (34.9%)	73 (35.1%)		
Coronary heart disease, *n* (%)				*χ*^2^ = 0.03	0.858^1^
No	214 (56.3%)	96 (55.8%)	118 (56.7%)		
Yes	166 (43.7%)	76 (44.2%)	90 (43.3%)		
Liver disease, *n* (%)				*χ*^2^ = 0.25	0.615^1^
No	354 (93.2%)	159 (92.4%)	195 (93.8%)		
Yes	26 (6.8%)	13 (7.6%)	13 (6.3%)		
Kidney disease, *n* (%)				*χ*^2^ = 1.99	0.159^1^
No	360 (94.7%)	166 (96.5%)	194 (93.3%)		
Yes	20 (5.3%)	6 (3.5%)	14 (6.7%)		
Cerebrovascular disease, *n* (%)				*χ*^2^ = 0.97	0.326^1^
No	262 (68.9%)	123 (71.5%)	139 (66.8%)		
Yes	118 (31.1%)	49 (28.5%)	69 (33.2%)		
Blood routine examinations					
WBC, median (Q1, Q3)	7.85 (6.30, 9.33)	7.70 (6.30, 9.20)	8.10 (6.38, 9.43)	*Z* = −0.99	0.323^2^
NEU, median (Q1, Q3)	2.70 (2.30, 3.30)	2.70 (2.20, 3.32)	2.80 (2.30, 3.30)	*Z* = −1.05	0.295^2^
EOS, median (Q1, Q3)	0.26 (0.13, 0.38)	0.26 (0.13, 0.37)	0.25 (0.12, 0.38)	*Z* = −0.60	0.547^2^
CRP, median (Q1, Q3)	8.60 (6.20, 13.20)	7.05 (5.50, 8.90)	11.55 (7.60, 17.07)	*Z* = −8.25	<0.001^2, *^
Immune function index					
CD3^+^CD4^+^T-cell count, median (Q1, Q3)	492.00 (431.00, 553.00)	522.50 (455.00, 566.50)	476.00 (419.75, 523.00)	*Z* = −5.20	<0.001^2, *^
CD3^+^CD8^+^ T-cell count, median (Q1, Q3)	484.50 (418.75, 544.25)	484.50 (424.75, 554.25)	485.00 (410.75, 537.25)	*Z* = −1.26	0.208^2^
CD4/CD8 ratio, median (Q1, Q3)	1.03 (0.88, 1.17)	1.02 (0.88, 1.18)	1.03 (0.88, 1.15)	*Z* = −1.02	0.306^2^
CD3-CD19^+^ B-cell count, median (Q1, Q3)	247.00 (189.00, 318.25)	235.00 (188.75, 310.00)	261.50 (191.25, 325.25)	*Z* = −1.33	0.184^2^
CD16^+^CD56^+^NK cell count, median (Q1, Q3)	387.00 (272.50, 494.25)	387.00 (272.50, 485.25)	386.00 (274.75, 508.25)	*Z* = −0.61	0.539^2^
Pulmonary function tests					
FEV1, median (Q1, Q3)	1.53 (1.33, 1.74)	1.53 (1.34, 1.78)	1.52 (1.32, 1.73)	*Z* = −0.94	0.347^2^
FEV1%Pred, median (Q1, Q3)	0.72 (0.67, 0.77)	0.73 (0.68, 0.78)	0.72 (0.67, 0.77)	*Z* = −1.47	0.142^2^
FVC, median (Q1, Q3)	1.89 (1.66, 2.22)	1.92 (1.66, 2.24)	1.87 (1.67, 2.18)	*Z* = −1.00	0.316^2^
FVC%Pred, median (Q1, Q3)	0.68 (0.62, 0.74)	0.68 (0.62, 0.75)	0.68 (0.62, 0.74)	*Z* = −0.86	0.392^2^
FEV1/FVC ratio, median (Q1, Q3)	0.80 (0.75, 0.85)	0.80 (0.75, 0.85)	0.80 (0.75, 0.85)	*Z* = −0.06	0.954^2^
FeNO, median (Q1, Q3)	13.00 (7.00, 19.00)	14.00 (8.00, 19.25)	12.00 (7.00, 18.00)	*Z* = −1.72	0.086^2^

^1^Pearson’s chi-squared test, ^2^Wilcoxon rank sum test, ^3^Fisher’s exact test. *p< 0.05, statistically significant. BMI, body mass index; BSI, Bronchiectasis Severity Index; FEV1, forced expiratory volume in 1 s; FVC, forced vital capacity; FeNO, fractional exhaled nitric oxide; WBC white blood cell; NEU, neutrophil; EOS, eosinophil; CRP, C-reactive protein; FEV1, forced expiratory volume in 1 s; FVC, forced vital capacity; FeNO, fractional exhaled nitric oxide.

### 3.2 Results of univariate logistic analysis

Univariate logistic regression analysis was performed for all variables. The results demonstrated that the following factors significantly associated with CBI in the lower respiratory tract of stable bronchiectasis patients (*p* < 0.05): age, BMI < 18.5 kg/m^2^, history of smoking, history of alcohol using, bronchiectasis duration (years), number of lobes affected ≥3, number of exacerbation in the prior year ≥3, history of hemoptysis in the prior year, mMRC score, BSI score, CRP, CD3^+^CD4^+^T-cell count<500 cells/μL. Comprehensive statistical outputs were presented in Table 2.

**Table 2.** Univariable logistic regression analysis of risk factors for chronic bacterial infection in stable bronchiectasis

Variable	Estimate	*S.E*	OR	95%CI	*P-*value
Sex					
female	Ref				
male	-0.30	0.21	0.74	0.49, 1.13	0.162
Age, years					
<50	Ref				
50-69	0.70	0.28	2.02	1.16, 3.51	0.013^*^
≥70	1.29	0.35	3.64	1.85, 7.17	<0.001^*^
BMI under 18.5(kg/m^2^)					
no	Ref				
yes	0.69	0.21	1.99	1.32, 2.99	0.001^*^
History of smoking					
no	Ref				
yes	0.60	0.29	1.83	1.05, 3.20	0.034^*^
History of alcohol using					
no	Ref				
yes	0.48	0.21	1.62	1.08, 2.44	0.020^*^
Disease duration, years	0.09	0.02	1.10	1.06, 1.14	<0.001^*^
Number of lobes affected					
1-2	Ref				
≥3	1.38	0.23	3.96	2.54, 6.17	<0.001^*^
Type of bronchiectasis					
cylindrical	Ref				
cystic	0.62	0.33	1.86	0.99, 3.52	0.055
both of above	0.72	0.25	2.05	1.27, 3.31	0.004^*^
Number of exacerbations in the prior year					
1-2	Ref				
≥3	1.76	0.29	5.80	3.31, 10.16	<0.001^*^
History of hemoptysis in the prior year					
no	Ref				
yes	1.36	0.29	3.91	2.24, 6.83	<0.001^*^
mMRC score	0.32	0.10	1.38	1.12, 1.68	0.002^*^
BSI score					
0-4	Ref				
5-8	0.88	0.22	2.41	1.56, 3.70	<0.001^*^
≥9	2.59	0.63	13.36	3.90, 45.86	<0.001^*^
Kidney disease					
no	Ref				
yes	0.69	0.50	2.00	0.75, 5.31	0.166
CRP	0.22	0.03	1.24	1.18, 1.31	<0.001^*^
CD3^+^CD4^+^T-cell count					
≥500 cells/μL	Ref				
<500 cells/μL	1.03	0.21	2.80	1.85, 4.26	<0.001^*^
CD3^+^CD8^+^T-cell count	-0.002	0.001	0.998	0.995, 1.001	0.177
CD4/CD8 ratio	-0.74	0.54	0.48	0.17, 1.37	0.170
CD3^-^CD19^+^B-cell count	0.002	0.001	1.002	0.999, 1.005	0.173
FEV1%Pred					
≥70	Ref				
<70	0.40	0.22	1.49	0.97, 2.28	0.066
FeNO	-0.03	0.02	0.97	0.94, 1.00	0.078

Note: *P<0.05, statistically significant*.* Ref, reference.

### 3.3 Screening of variables with LASSO regression

**Figure 1:**
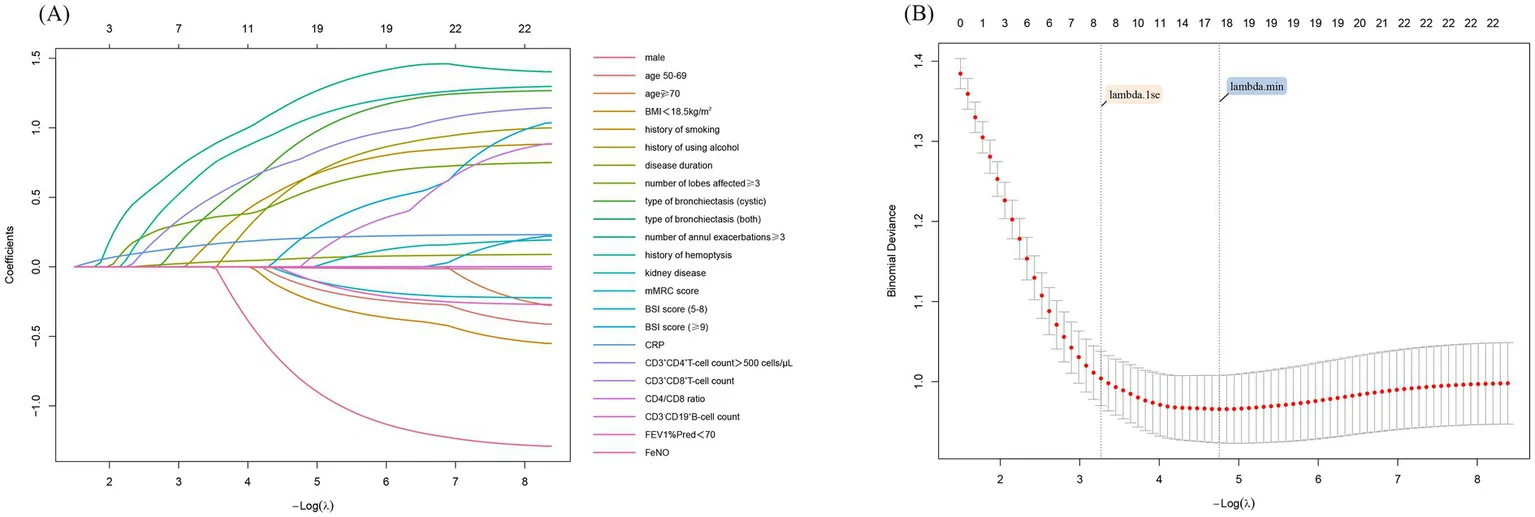
The variable filtering process of the Lasso regression for variables with *p* > 0.2 in univariate logistic regression analysis. **(A)** Lasso regression coefficient profiles showing the dynamic trajectories of candidate variable coefficients across different *lambda* values. **(B)** The selection of optimal parameters (*lambda*) by ten-fold cross-validation, in which dotted vertical lines were drawn at the optimal values by using the minimum criteria and limits defined by 1 standard deviation.

Twenty variables with *p* < 0.2 in univariate logistic analysis were incorporated into the LASSO regression to screen potential predictors of CBI. In the LASSO model, the optimal regularization parameter (*λ*) was determined using 10-fold cross-validation. Plotted vertical lines were drawn at the optimal values by using the minimum criteria and the 1 standard deviation of the minimum criteria. The LASSO regression recommended eight predictors for CBI from the potential 20 variables (Figure 1). These predictors were history of smoking, disease duration (years), number of lobes affected ≥3, type of bronchiectasis, number of exacerbation in the prior year ≥3, history of hemoptysis in the prior year, CRP, CD3^+^CD4^+^T-cell count <500 cells/μL.

### 3.4 Results of multivariate logistic analysis

Based on LASSO regression results, literature evidence, and clinical expertise, seven variables were incorporated multivariate logistic analysis, including history of smoking, disease duration (years), number of lobes affected ≥3, number of exacerbation in the prior year ≥3, history of hemoptysis in the prior year, CRP, CD3^+^CD4^+^T-cell count <500 cells/μL.

The multivariate logistic regression analysis demonstrated that seven indicators were identified as significant independent risk factors for CBI in patients with stable bronchiectasis (Table 3), including history of smoking, disease duration, number of lobes affected ≥3, number of exacerbation in the prior year ≥3, history of hemoptysis in the prior year, CRP, CD3^+^CD4^+^T-cell count <500 cells/μL. Disease duration was an independent risk factor for CBI (OR, 1.07; 95% CI: 1.02, 1.12; *P*, 0.009). Each additional year of disease duration corresponded to a 7% increase in the risk of CBI. Patients with a smoking history exhibited a 2.22-fold higher risk of CBI compared with non-smokers (OR, 2.22; 95% CI: 1.10, 4.49; *P* = 0.026). Bronchiectasis involving more than three lung lobes increased the risk of CBI (OR, 1.79; 95% CI: 1.01, 3.19; *P*, 0.047), number of exacerbation in the prior year ≥3 (OR, 3.22; 95% CI: 1.63, 6.35; *P*﹤0.001) and history of hemoptysis in the prior year (OR, 3.85; 95% CI: 1.95, 7.60; *P*﹤0.001) were strong independent predictors for CBI. Elevated CRP level was positively correlated with the risk of CBI (OR, 1.25; 95% CI: 1.17, 1.33; *P*﹤0.001). Additionally, CD3^+^CD4^+^T-cell count <500 cells/μL was an independent risk factors for CBI (OR, 2.24; 95% CI: 1.32, 3.78; *P*, 0.003).

**Table 3.** Multivariate logistic regression analysis of risk factors for chronic bacterial infection in stable bronchiectasis

Variable	Estimate	*S.E*	OR	95% CI	*P*-value
Disease duration, years	0.06	0.02	1.07	1.02, 1.12	0.009^*^
History of smoking					
no	Ref				
yes	0.80	0.36	2.22	1.10, 4.49	0.026^*^
Number of lobes affected					
1-2	Ref				
≥3	0.59	0.29	1.79	1.01, 3.19	0.047^*^
Number of exacerbations in the prior year					
1-2	Ref				
≥3	1.17	0.35	3.22	1.63, 6.35	<0.001^*^
History of hemoptysis in the prior year					
no	Ref				
yes	1.35	0.35	3.85	1.95, 7.60	<0.001^*^
CRP	0.22	0.03	1.25	1.17, 1.33	<0.001^*^
CD3^+^CD4^+^ T-cell count					
≥500 cells/μL	Ref				
<500 cells/μL	0.81	0.27	2.24	1.32, 3.78	0.003^*^

Note: *P<0.05, statistically significant*.* Ref, reference.

### 3.5 Nomogram model establishment and validation

**Figure 2:**
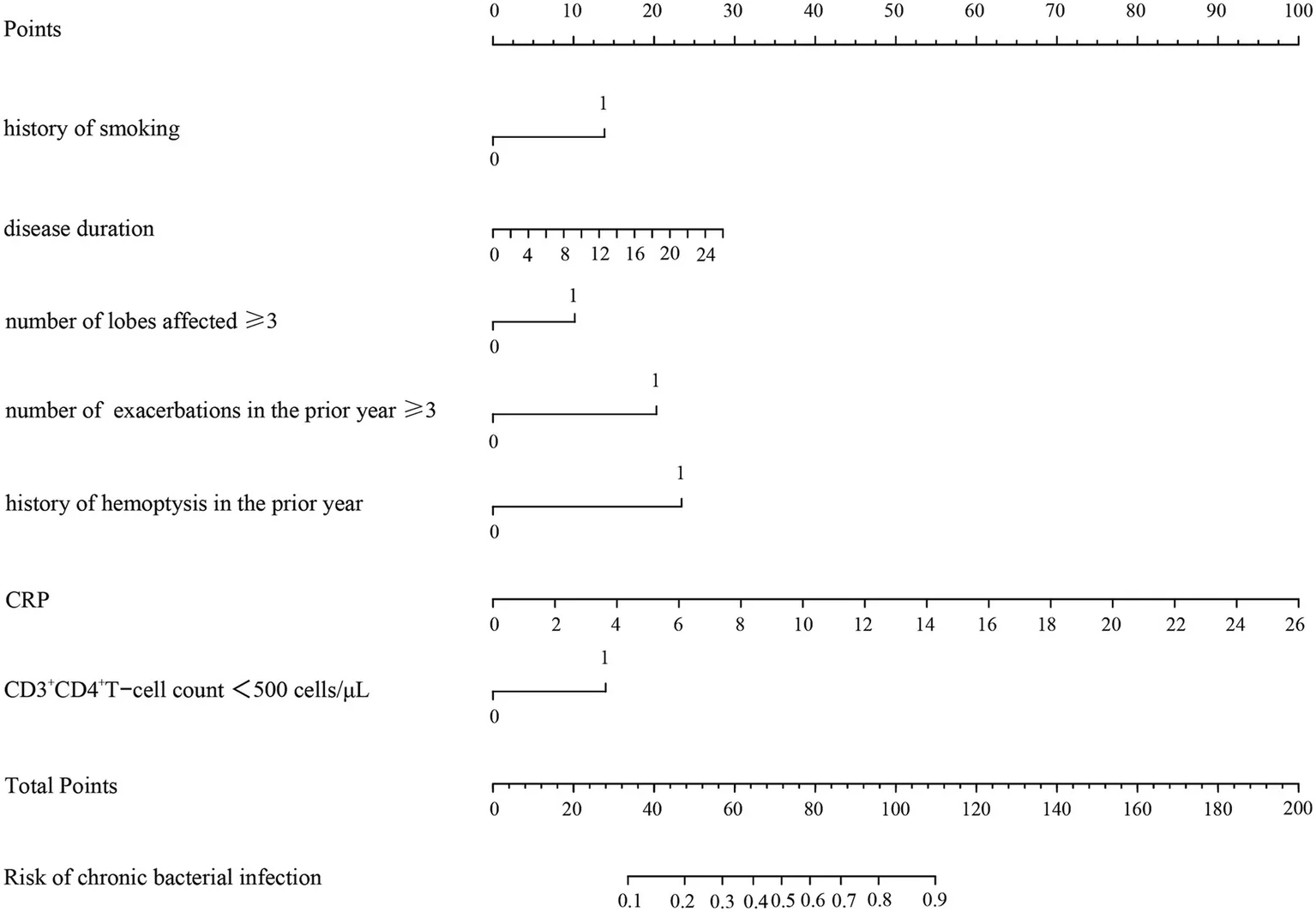
The nomogram model incorporates seven predictors screened by multivariate logistic regression analysis to estimate the risk of chronic bacterial infection in patients with stable bronchiectasis. Each variable is assigned a score according to its contribution to the model. The total score is calculated by summing all individual scores, and corresponds to the predicted risk.

A nomogram prediction model (Figure 2) was constructed by incorporating the seven optimal predictors identified in the multivariate logistic analysis. In the nomogram, each variable’s options corresponded to respective scores, and the total score was obtained by summing those of all seven variables. At the bottom of Figure 2, predictions for the probabilities corresponding to different total scores were given. The higher the total score, the greater the risk of CBI.

**Figure 3:**
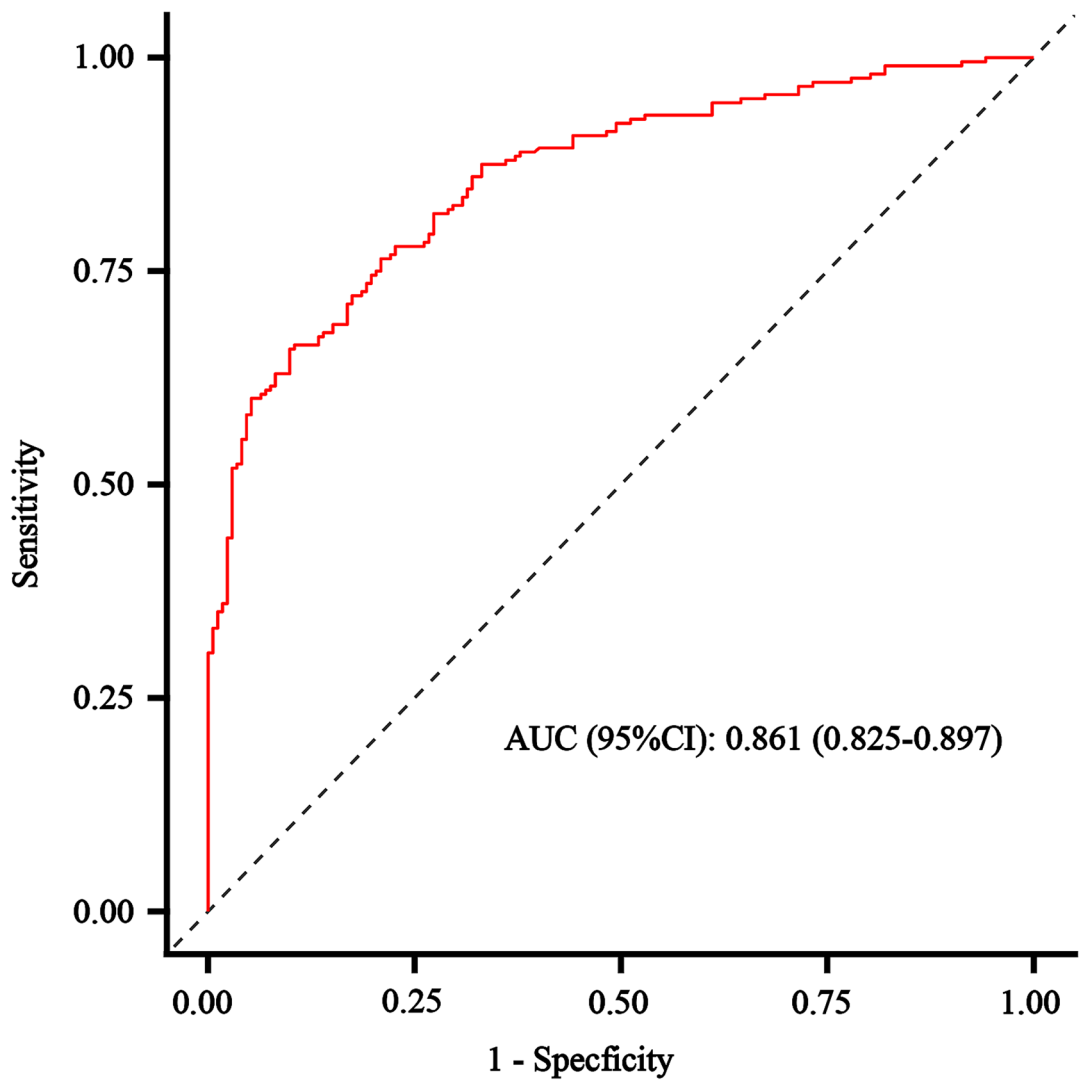
ROC curves of the nomogram model for chronic bacterial infection of stable bronchiectasis. The area under ROC curve is utilized to judge the advantages and disadvantages of nomogram model.

**Figure 4:**
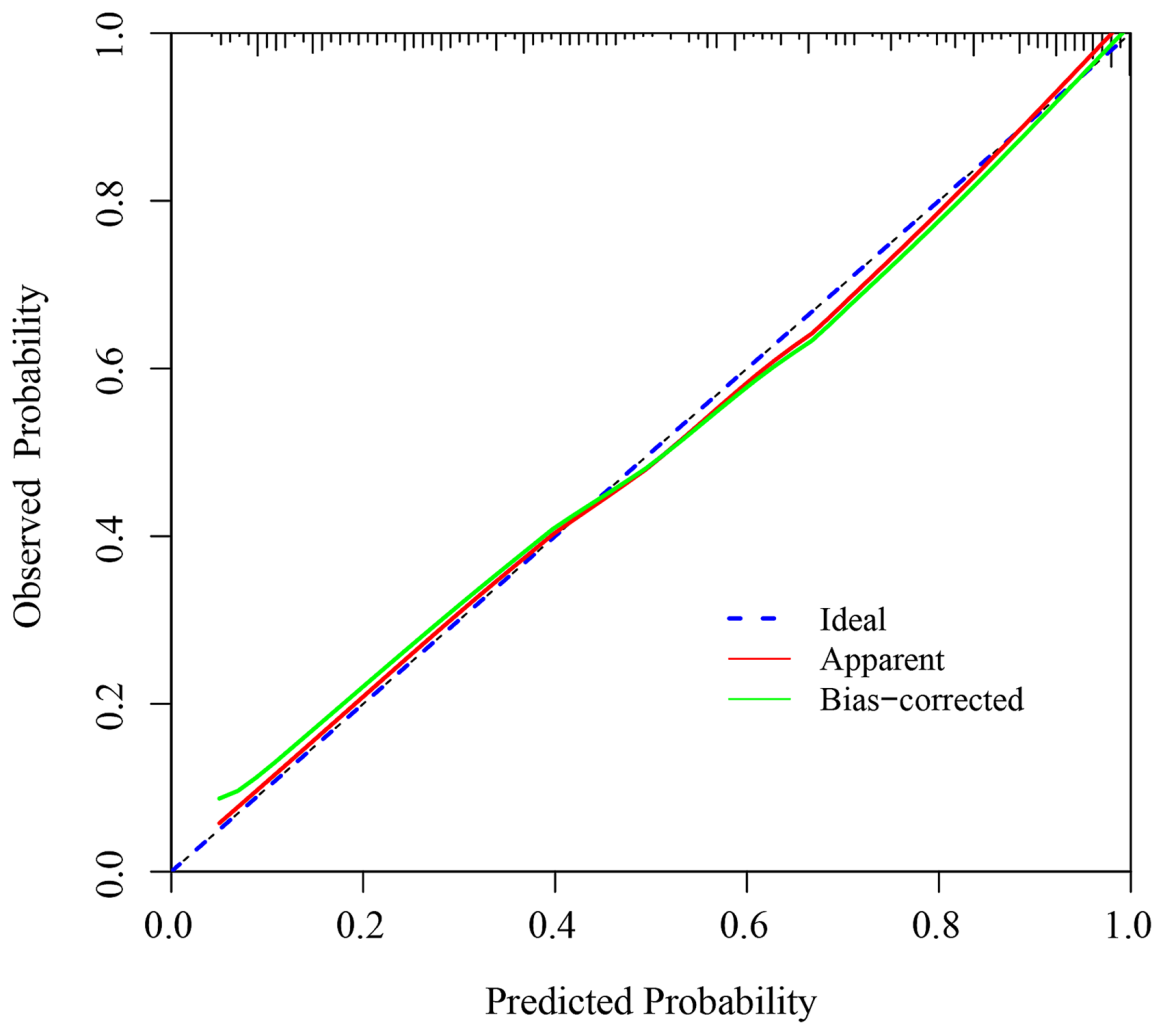
Calibration plot of the nomogram model for chronic bacterial infection in stable bronchiectasis. The diagonal 45-degree line indicates perfect prediction. High consistency between the apparent curve, bias-corrected curve and ideal curve indicates good calibration performance of the model.

ROC analysis of the nomogram revealed that AUC was 0.861 (95% CI: 0.825–0.897; Figure 3). It suggested that the nomogram has excellent discriminant accuracy. At the maximum Youden index of 0.560, the model achieved a sensitivity of 65.9% and specificity of 90.1%. The calibration curve showed bias-correction and an apparent curve similar to the ideal line (Figure 4), demonstrating a strong consistency between actual observation and prediction. The Hosmer-Lemeshow test proved the nomogram model’s fit was good (*p* = 0.794).

**Figure 5:**
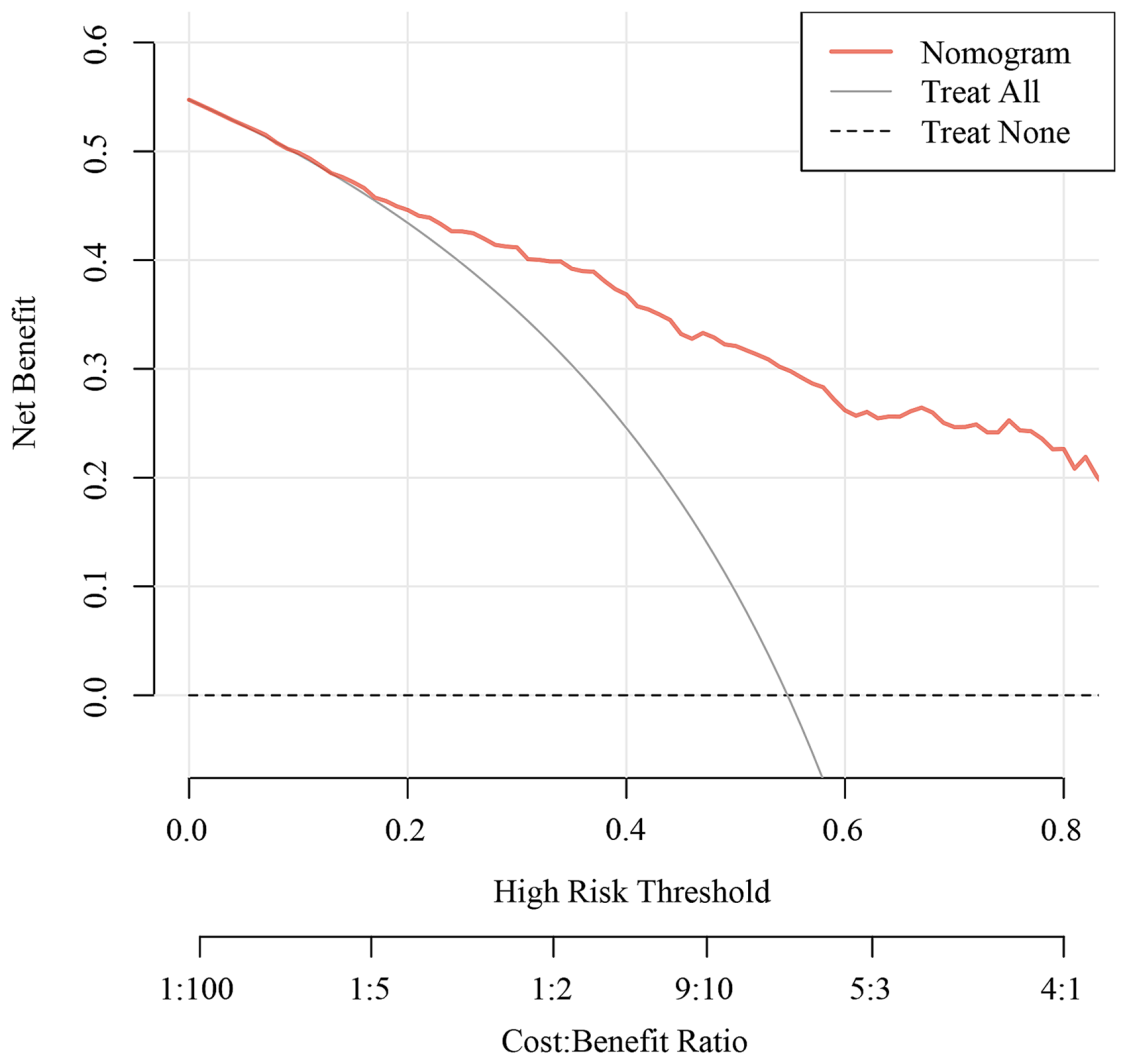
Decision curve analysis of the nomogram model. The net benefit calculated by adding true positive and minus the false positive corresponds to the measurement of Y-axis. X-axis represents the threshold probability.

Additionally, DCA was performed using CBI as the outcome variable and nomogram-derived risk predictions as the test variable (Figure 5). The nomogram curve remained above the all-curve and none-curve across most threshold probability ranges, demonstrating a significant positive net benefit. It indicated its great clinical practical value in predicting CBI of stable bronchiectasis.

## 4 Discussion

In recent years, the incidence and prevalence of bronchiectasis have gradually increased worldwide, making bronchiectasis an increasingly serious public health problem, imposing a heavy burden on patients’ quality of life and the medical system. Recurrent bacterial infections of the lower respiratory tract represent a key pathophysiological process in bronchiectasis and serve as a major driving force underlying disease progression (28). Therefore, constructing a clinical prediction model for CBI in patients with stable bronchiectasis to identify high-risk individuals is of great clinical significance for the early intervention and management of patients with bronchiectasis.

During the stable phase of bronchiectasis, PPMs in the lower airways exist as commensals, in a state of asymptomatic and tolerated equilibrium with host immunity. When host immunity declines or PPMs undergo immune escape, this homeostasis is disrupted, leading to massive proliferation of pathogenic bacteria that transition to a pathogenic state. These pathogens stimulate the body to release inflammation-related factors, perpetuating the vicious vortex and resulting in exacerbations of bronchiectasis. During exacerbations, antibiotic intervention reduces the bacterial burden of colonized PPMs in the lower airways, which alleviates the body’s inflammatory response, and the host immunity gradually restores, thereby re-establishing homeostasis (29). This cycle recurs continuously, and given that the stable phase constitutes the majority of the bronchiectasis disease course, prevention during the stable phase is more crucial than treatment during the acute phase.

Currently, microbiological detection methods for lower respiratory tract specimens include sputum culture, antigen detection, antibody detection, polymerase chain reaction (PCR), and next-generation sequencing (NGS). Sputum culture, the gold standard for detecting respiratory pathogens, can effectively distinguish different pathogens. As one of the traditional detection methods, although sputum is susceptible to oral contamination, it has the advantages of low detection cost and simple operation, making it suitable for most hospitals and primary health institutions and enabling coverage of a larger population. Meanwhile, subsequent drug sensitivity tests can help understand the sensitivity of pathogens to antibiotics and guide clinical medication (30). Antigen and antibody detection is a method that uses known pathogen antigens or antibodies for detection. It is characterized by simple operation and ease of use, but is prone to false negatives, meaning that a negative result cannot rule out infection with the pathogen (31). PCR is a molecular technique that detects pathogens by identifying specific genetic sequences. It offers high accuracy and sensitivity, but it does not differentiate between viable and non-viable microorganisms. This limitation makes it challenging to distinguish between active infection and past exposure (32). NGS is a high-throughput technology capable of performing comprehensive nucleic acid analysis directly. Although it offers advantages such as shorter turnaround time, high accuracy, and broad detection coverage, its relatively high cost remains a limitation. Therefore, NGS may serve as a complementary tool rather than a replacement for conventional biological detection methods (33, 34). We mainly used sputum culture as the criterion for judging chronic bacterial infections in patients with bronchiectasis.

Among the 380 enrolled patients with stable bronchiectasis, female individuals accounted for a larger proportion than males. This demographic characteristic was consistent with findings from previous studies (10, 35–37). In this study, the rate of CBI was 54.74% (208/380). The pathogen with the highest detection rate was *Pseudomonas aeruginosa*, followed by *Haemophilus influenzae*, which is consistent with the results of EMBARC cohort (38). In the BE-China cohort and Indian cohort, PA represented the most common pathogen, and the second most frequently isolated microbe differed across these regions (37, 39). This variation could be explained by geography, socioeconomic conditions, and ethnic factors. About 25% of the patients with bronchiectasis are likely to develop chronic PA infection (40). Chronic PA infection is closely associated with more frequent exacerbations, hospitalizations, worse lung function, poorer quality of life, more severe pulmonary imaging findings, and higher mortality in patients with bronchiectasis in a stable state (41, 42). Specifically, PA primarily activates the host inflammatory response and disrupts the airway epithelial barrier through the production of virulence factors (such as LPS, flagella, and elastase) and the formation of biofilms, leading to chronic infection and microbiome imbalance, which drives the vicious vortex of bronchiectasis (43). Similarly, *Haemophilus influenzae* can establish chronic infection in the human body through adhesion, intracellular invasion, and biofilm formation. Persistent infection further exacerbates airway inflammation and lung structural damage, forming a vicious vortex (44). A study shown that *Haemophilus influenzae* infection was an independent predictor of exacerbations, and could lead to more frequent exacerbations (45).

The pathophysiological process of the “vicious vortex” of bronchiectasis constitutes the fundamental cause of persistent symptoms, progressive decline in pulmonary function, and deterioration of quality of life in patients with bronchiectasis (46). A prolonged disease duration implies long-term exposure to a vicious cycle of chronic inflammation, recurrent infection, impaired mucus clearance, and progressive destruction of lung architecture, thereby leading to more severe cumulative lung injury. Previous study had reported a positive correlation between disease duration and colonization by PPMs (47). A longer disease duration was closely associated with more severe structural abnormalities of the lung (48). Our results demonstrated that a longer disease duration of bronchiectasis correlated with an elevated risk of CBI in stable bronchiectasis. Accordingly, regular sputum culture testing serves as a vital measure to early identify CBI and implement timely interventions in patients with long-standing bronchiectasis.

The number of lobes affected is an important component of both the BSI and E-FACED scores. It can not only reflect the extent and severity of bronchiectasis, but also predict the risks of future mortality, hospitalization, exacerbation, and quality of life (15, 49). With an increasing number of lobes affected, structural lung damage becomes more severe, airway clearance function declines progressively, and the risk of CBI rises accordingly (6). Multi-lobar bronchiectasis was associated with greater disease severity and more frequent exacerbations (50). A retrospective analysis identified involvement of more than two lung lobes as an independent significant risk factor for massive hemoptysis in patients with bronchiectasis (OR = 4.347, 95%CI: 1.960–9.638; OR = 2.787, 95%CI: 1.055–7.363) (51). Our analysis indicated that three or more affected lung lobes were predictive of chronic bacterial infection (CBI). Therefore, it is important for predicting the risk of CBI and disease severity to regular follow-up chest computed tomography (CT).

Exacerbations is the critical event in the clinical course of bronchiectasis. Increased frequency of acute exacerbations was associated with greater economic burden among patients with bronchiectasis (52). Frequent exacerbators were defined as those experiencing three or more exacerbations per year (10). A study demonstrated that patients with frequent exacerbations have a 2-fold higher mortality rate compared with those without exacerbations (53). Previous research indicated that three or more exacerbations in the prior year exhibited optimal predictive sensitivity and specificity for disease progression in bronchiectasis. It was correlated with higher risks of hospitalization and mortality, as well as poorer quality of life (45). Bacterial infection is widely regarded as a key trigger for exacerbations of bronchiectasis. Using sensitive PCR assays, bacteria were detected in 86% of sputum samples obtained during exacerbations (54). In addition, acute exacerbations act as important drivers of the “vicious vortex” in bronchiectasis, which can further aggravate structural lung damage and increase the risk of bacterial infection (55). According to our findings, patients with three or more exacerbations in the prior year were at higher risk of chronic bacterial infection. Guidelines from the European Respiratory Society identified three or more exacerbations per year as an indication for initiating antibiotic prophylaxis (13). Therefore, bronchiectasis patients with frequent exacerbations require early prophylactic antibiotic therapy to prevent the development of chronic bacterial infection.

A United States study reported that approximately 23% of patients with bronchiectasis had a history of hemoptysis (56). New-onset or aggravated hemoptysis in bronchiectasis patients may be precipitated by respiratory infections (57). A study demonstrated that hemoptysis correlates with an infective phenotype of bronchiectasis (50). These suggested its potential role as a clinical indicator of lower respiratory tract infections. An international consensus by bronchiectasis experts identified hemoptysis as a defining symptom of acute exacerbations (14). Among adult patients with bronchiectasis, the mortality rate secondary to hospitalization for hemoptysis was approximately 4.5% (58). Studies have verified that the incidence of severe and recurrent hemoptysis is 2.87-fold higher in patients with *Pseudomonas aeruginosa* infection than in uninfected counterparts (59). Although prior studies have demonstrated associations of hemoptysis with acute exacerbations and respiratory infections, its link to chronic bacterial infection in bronchiectasis patients is still undefined. Our study demonstrated that bronchiectasis patients with a history of hemoptysis in the prior year had a 3.85-fold higher risk of developing CBI than those without hemoptysis. Therefore, regular sputum culture for those with hemoptysis can be meaningful in guiding and identifying exacerbations of bronchiectasis.

Cigarette smoking is an established risk factor for pulmonary diseases including COPD, lung cancer, and tuberculosis. Evidence indicated that smoking history associates with earlier onset of bronchiectasis in young adults, and elevated mortality risk (60). Through multiple pathological mechanisms, including induction of structural alterations, suppression of immune function, impairment of mucociliary clearance, and disruption of protease-antiprotease balance, cigarette smoking exacerbates inflammatory responses, and increases susceptibility to recurrent respiratory infections and chronic bacterial colonization (61–63). Cigarette smoking impairs airway mucosal integrity, increases pathogen virulence, and promotes biofilm formation, thereby facilitating microbial adhesion within the respiratory tract (64). Smoking history was an independent risk factor for post-operative pneumonia in bronchiectasis (OR = 8.735), and correlated with increased mortality, frequent exacerbations, and reduced quality of life (65, 66). Notably, smokers with preserved lung function exhibited higher risks of suspected bronchiectasis and mortality (67). Currently, smoking was known to be closely associated with the development and progression of pulmonary diseases such as COPD and lung cancer. However, its correlation with bronchiectasis remains unclear. Our study identified a smoking history as an independent risk factor for chronic bacterial colonization in patients with bronchiectasis. Therefore, it is recommended that clinicians regularly perform sputum culture testing in bronchiectasis patients with a history of smoking to facilitate early identification and intervention, thereby preventing acute exacerbations.

Bronchiectasis is recognized as a complex inflammatory disorder characterized by not only persistent airway inflammation but also systemic inflammatory responses (68). Elevated systemic inflammatory biomarkers such as CRP serve as predictors of bacterial infection and correlate with disease severity (57). A prospective single-center study found that serum CRP > 0.35 mg/dL predicts heightened risks of acute exacerbations and hospitalizations, suggesting CRP can directly reflect the systemic inflammatory status in patients with stable bronchiectasis (69). Furthermore, elevated CRP levels were strongly correlated with increased exacerbation frequency and served as an independent predictor of frequent exacerbations (70). We observed that CRP levels were positively correlated with the risk of CBI in patients with bronchiectasis. Therefore, elevated CRP levels should raise suspicion of potential bacterial infection, warranting enhanced sputum culture screening and early implementation of anti-infection strategies to prevent disease progression in bronchiectasis.

Impaired host immune responses constitute a fundamental pathophysiological mechanism in bronchiectasis (71). T lymphocytes constitute the core component of adaptive immunity and reflect the host’s cellular immune capacity (72, 73). Immune dysfunction drives bronchiectasis progression by diminishing pathogen clearance capacity (6). It increases susceptibility to lower respiratory tract colonization, and accelerates disease deterioration (74). A study has indicated that persistent infection may exhaust T-cell immune function (75). Our study found that the count of CD3^+^CD4^+^T lymphocyte cell < 500 cells/μL was an independent risk factor for CBI in patients with bronchiectasis. Additionally, immunonutritional support (such as vitamin D, zinc) may enhance immune competence, reducing infection risk (76).

## 5 Conclusion

This study identified risk factors for CBI of lower respiratory tract in patients with stable bronchiectasis and developed a reasonably accurate risk prediction model. The establishment of this model can help raise clinical awareness of the necessity of including sputum culture as a routine test for bronchiectasis, screen populations at high risk of chronic bacterial infection, facilitate the timely initiation of antibiotics, prevent acute exacerbations, and control disease progression. Although the model demonstrated good discrimination and accuracy, it has several limitations: (1) This study utilized collected data from a single center, which may contain certain confounding factors; (2) The sample size was relatively small. It is recommended that future multicenter longitudinal studies with expanded sample sizes be conducted to further optimize this risk model. Through clinical practice, our research team has increasingly recognized the importance of early antibiotic intervention in patients with bronchiectasis who have chronic bacterial infection, as it may reduce the frequency of acute exacerbations and improve quality of life. Therefore, based on this study, we will conduct further research on related treatment strategies to provide new approaches for controlling the progression of bronchiectasis.

## Data Availability

The datasets presented in this article are available from the corresponding author upon reasonable request. Requests to access the datasets should be directed to Lei Qiu, dr_qiulei@shutcm.edu.cn.
